# Association of Influenza Vaccination With SARS-CoV-2 Infection and Associated Hospitalization and Mortality Among Patients Aged 66 Years or Older

**DOI:** 10.1001/jamanetworkopen.2022.33730

**Published:** 2022-09-28

**Authors:** Seyed M. Hosseini-Moghaddam, Siyi He, Andrew Calzavara, Michael A. Campitelli, Jeffrey C. Kwong

**Affiliations:** 1 ICES, Toronto, Ontario, Canada; 2 Ajmera Transplant Centre, University Health Network, Toronto, Ontario, Canada; 3 Division of Infectious Diseases, Department of Medicine, University of Toronto, Toronto, Ontario, Canada; 4 Public Health Ontario, Toronto, Canada; 5 Department of Family and Community Medicine, University of Toronto, Toronto, Ontario, Canada; 6 Dalla Lana School of Public Health, University of Toronto, Toronto, Ontario, Canada; 7 Centre for Vaccine Preventable Diseases, University of Toronto, Toronto, Ontario, Canada

## Abstract

**Importance:**

Vaccine effectiveness studies have rarely implemented strategies to reduce the healthy vaccinee bias arising from differences in health care–seeking behavior between vaccinated and unvaccinated individuals. Although previous observational studies suggest that influenza vaccination is associated with a reduced risk of SARS-CoV-2–associated outcomes, the healthy vaccinee bias may have led to overestimating the vaccination effect.

**Objective:**

To estimate the association between influenza vaccination and SARS-CoV-2–associated outcomes.

**Design, Setting, and Participants:**

This cohort study was conducted over 2 consecutive influenza vaccination campaigns (2019-2020 and 2020-2021), owing to the substantial COVID-19 burden and the greater validity of influenza vaccination data in the studied age group. The study population included community-dwelling adults aged 66 years or older in Ontario, Canada.

**Exposure:**

Influenza vaccination for a given season.

**Main Outcomes and Measures:**

The outcomes of interest included SARS-CoV-2 infection, SARS-CoV-2–associated hospitalization, SARS-CoV-2–associated death, and a composite of SARS-CoV-2–associated hospitalization or death. Cox proportional hazards models were used to measure the association between influenza vaccination and SARS-CoV-2–associated outcomes, censoring individuals who moved into long-term care, received COVID-19 vaccines, or died before the observation period end date. Primary care periodic health examinations (PHEs) were explored as a negative tracer exposure (ie, no association expected with SARS-CoV-2 outcomes) and as an effect modifier of the association between influenza vaccination and SARS-CoV-2 outcomes.

**Results:**

Of 2 922 449 individuals aged 66 years or older (54.2% female) living in Ontario, 2 279 805 were included in the study. Among these, 1 234 647 (54.2%) were female and 1 045 158 (45.8%) were male; their mean (SD) age was 75.08 (7.21) years. Those who had received influenza vaccination exhibited a lower incidence of SARS-CoV-2 infection than unvaccinated individuals for the 2019-2020 cohort (adjusted hazards ratio [aHR], 0.78; 95% CI, 0.73-0.84) and the 2020-2021 cohort (aHR, 0.76; 95% CI, 0.74-0.78). This association was also observed for SARS-CoV-2–associated hospitalization or death (2019-2020: aHR, 0.83; 95% CI, 0.74-0.92; 2020-2021: aHR, 0.66; 95% CI, 0.63-0.70). Similarly, undergoing a PHE was also associated with a lower incidence of SARS-CoV-2 infection (aHR, 0.85; 95% CI, 0.78-0.91) and SARS-CoV-2–associated hospitalization or death (aHR, 0.80; 95% CI, 0.70-0.90), and modified the association between influenza vaccination and SARS-CoV-2 infection for vaccinated individuals who underwent PHE (aHR, 0.62; 95% CI, 0.52-0.74) and for vaccinated individuals who did not undergo PHE (aHR_,_ 0.81; 95% CI, 0.76-0.87), and also SARS-CoV-2–associated hospitalization or death in vaccinated individuals who underwent PHE (aHR, 0.66; 95% CI, 0.49-0.88) and vaccinated individuals who did not undergo PHE (aHR, 0.85, 95% CI, 0.76-0.95).

**Conclusions and Relevance:**

The findings of this cohort study suggest that undergoing a PHE may at least partially modify the association between influenza vaccination and SARS-CoV-2–associated outcomes in individuals aged 66 years or older, providing evidence of the healthy vaccinee bias that may affect vaccine effectiveness studies.

## Key Points

**Question:**

Is influenza vaccination associated with SARS-CoV-2 infection and related outcomes, such as hospitalization and mortality in individuals aged 66 years or older?

**Findings:**

In this cohort study of 2 279 805 patients, influenza vaccination was found to be associated with a 22% to 24% lower risk of SARS-CoV-2 infection; however, undertaking a periodic health examination (PHE) was also associated with a 15% lower risk of SARS-CoV-2 infection. The negative association between influenza vaccination and SARS-CoV-2–related outcomes differed considerably when the analysis was stratified by the history of PHE in the preceding year.

**Meaning:**

The findings of this study suggest that a PHE may at least partially modify the association between influenza vaccination and SARS-CoV-2–associated outcomes in individuals aged 66 years or older owing to the healthy vaccinee bias.

## Teaser

This cohort study assesses whether influenza vaccination is associated with SARS-CoV-2 infection and related outcomes such as hospitalization or mortality in individuals aged 66 years or older.

## Introduction

A novel coronavirus now known as severe acute respiratory syndrome coronavirus 2 (SARS-CoV-2) is considered to have caused the COVID-19 pandemic. The pandemic response has placed a great emphasis on vaccination.

Although clinical trials have demonstrated the efficacy of novel vaccines specifically targeting the SARS-CoV-2 spike protein,^1^ observational studies suggest that influenza vaccines may confer some protection against SARS-CoV-2–related outcomes.^2,3,4,5^ A meta-analysis of 16 observational studies involving 290 327 participants showed that the receipt of influenza vaccine was associated with a lower risk of SARS-CoV-2 infection (pooled adjusted odds ratio [OR], 0.86; 95% CI, 0.81-0.91).^2^ Data from Brazil on more than 50 000 patients hospitalized because of COVID-19 demonstrated that influenza vaccination was associated with a 7% lower odds of admission for intensive care treatment, 17% lower odds of requirement of mechanical ventilation, and 16% lower odds of death.^3^ Ecological analyses of large-scale data from the US and Italy have observed an inverse association between influenza vaccine coverage and COVID-19 mortality rates.^4,5^ Some potential mechanisms to support the biological plausibility for the inverse association observed between influenza vaccination and SARS-CoV-2–related outcomes have been offered, including induction of innate immunity,^6^ trained immunity,^7^ vaccine-associated viral interference,^2^ “original antigenic sin,”^8^ heterologous effects,^9,10^ and common pathogenesis features.^11^

Because receipt of vaccines is generally voluntary and is largely sought by motivated, health-conscious individuals who are more likely to observe health-promoting behaviors (eg, hand hygiene),^12,13^ vaccine effectiveness studies are susceptible to the “healthy vaccinee bias,” which may exaggerate the apparent protective effects of vaccines.^14^

This study assessed the associations between influenza vaccination and SARS-CoV-2 infection and related outcomes, such as hospitalization and mortality. We examined primary care periodic health examinations (PHEs) as a negative tracer exposure (ie, no association with SARS-CoV-2–associated outcomes expected) and as an effect modifier.^15^

## Methods

### Study Design, Setting, and Population

We performed a population-based cohort study using administrative health care data. These data sets were linked using encoded identifiers and analyzed at ICES, a not-for-profit research institute in Ontario, Canada. Data utilization and analysis have been authorized under Section 45 of Ontario’s Personal Health Information Protection Act, which does not require ethics board approval. Our findings were reported according to the Strengthening the Reporting of Observational Studies in Epidemiology (STROBE) reporting guideline (eTable 1 in the Supplement).

The study population included 2 annual cohorts (2019-2020 and 2020-2021) of all adults aged 66 years or older. The start date of the follow-up for the 2019-2020 cohort was October 1, 2019, and included influenza vaccinations received until March 31, 2020, while the follow-up for the 2020-2021 cohort started on October 1, 2020, and included influenza vaccinations received until March 31, 2021. The maximum follow-up dates for the 2 cohorts were September 30, 2020, and April 30, 2021, respectively. All individuals had free and universal access to influenza vaccines, hospital and physician care, and SARS-CoV-2 testing using real-time reverse transcription–polymerase chain reaction (RT-PCR). We excluded non-Ontario residents, individuals ineligible for the Ontario Health Insurance Plan (OHIP) and those with no recorded sex or date of birth, persons with no contact with the health care system in the 3 years prior to follow-up, and people living in long-term care (eTable 2 in the Supplement).

### Exposure and Outcomes

The exposure of interest was influenza vaccination for a given season, treated as a time-dependent covariate, 14 days or more before a positive SARS-CoV-2 test result. The primary outcome was a positive SARS-CoV-2 RT-PCR test result.^16^ Patients with indeterminate results were not considered positive. The secondary outcomes included SARS-CoV-2–associated hospitalizations and SARS-CoV-2–associated deaths, defined as all-cause hospitalizations and all-cause deaths within 14 days and 30 days, respectively, following a positive SARS-CoV-2 test result, and a composite of SARS-CoV-2–associated hospitalization or death. Because these outcomes are contingent on getting tested for SARS-CoV-2, we also examined the likelihood of undergoing SARS-CoV-2 testing in vaccinated and unvaccinated individuals to ensure that the observed associations are not simply due to differences in the likelihood to seek testing.

### Data Sources

We used the COVID-19 Integrated Testing Database, a comprehensive data set created by ICES that includes all available provincial SARS-CoV-2 test results and public health surveillance data, to identify patients with confirmed SARS-CoV-2 infection. The Ontario Registered Persons database, which contains vital statistics information for provincial residents, was used to ascertain demographic characteristics and date of death. Clinical and administrative data from inpatient admissions to all acute care hospitals in Ontario were obtained from the Canadian Institute for Health Information’s Discharge Abstract Database. The OHIP billing claims database was used to collect service and diagnostic information from physician-based care interactions, including influenza vaccination in physician offices. We used the Ontario Drug Benefit database to identify influenza vaccination by pharmacists. The definitions and data sources used for each variable are provided in eTable 3 in the Supplement.

### Measures

The baseline variables included sex, age group (ie, 66-74, 75-84, and ≥85 years), public health unit (PHU) region, rural vs urban residence, and census dissemination area–level measures of household income, household density, apartment building density, recent immigration, self-identification as visible minority, uncoupled adults, educational attainment, and essential work status. Comorbidities included asthma, chronic obstructive pulmonary disease, diabetes, active cancer, chronic kidney disease, advanced liver disease/cirrhosis, autoimmune disorder, immunocompromised status (ie, patients with HIV infection, solid organ or stem cell transplant recipients, individuals receiving immunosuppressive therapy), rheumatoid arthritis, inflammatory bowel disease, frailty, dementia, hypertension, ischemic heart disease, congestive heart failure, and transient ischemic attack/stroke. Because we hypothesized that health care use may likely be associated with increased access to testing and may also be a marker for comorbidities, we measured health care use by the number of acute-care hospital admissions in the past 3 years and physician office visits in the preceding year.

### Statistical Analysis

#### Main Analyses

We compared the baseline characteristics between vaccinated and unvaccinated individuals in each cohort. We used multivariable Cox proportional hazards models to estimate cause-specific hazard ratios (HRs) (reported with 95% CIs) for the association between influenza vaccination and SARS-CoV-2–related outcomes. Influenza vaccination was modeled as a binary time-varying covariate. Individuals were censored if they died, moved into long-term care, or received a COVID-19 vaccine.

In all analyses, we calculated adjusted HRs (aHRs) after adjusting for age and sex, and subsequently fully adjusted HRs accounting for age, sex, comorbidities, past health care use, receipt of the previous-year influenza vaccination, neighborhood income, rurality, PHU region, and census variables. To examine the association between influenza vaccination and having an RT-PCR test result for SARS-CoV-2, we used logistic regression to calculate adjusted odds ratios (AORs), accounting for the same factors.

#### Post Hoc Analyses

To examine the presence of the healthy vaccinee effect, we evaluated the association between having a PHE in 2018-2019 and SARS-CoV-2–related outcomes in 2019-2020, essentially treating PHE as a negative tracer exposure because there is no biological plausibility to expect that having a PHE should reduce the risk of SARS-CoV-2 infection and related outcomes. We subsequently examined the association between influenza vaccination and SARS-CoV-2–related outcomes stratified by PHE, thereby treating it as a potential effect modifier.

For the 2019-2020 cohort, we evaluated the influence of repeated influenza vaccination by comparing individuals who did not receive influenza vaccination in either 2018-2019 or 2019-2020 (NN; reference group) vs those who received influenza vaccination in both 2018-2019 and 2019-2020 (YY), those who received influenza vaccination in 2018-2019 but not in 2019-2020 (YN), and those who did not receive the vaccination in 2018-2019 but received it in 2019-2020 (NY). We did not include the 2020-2021 cohort in this analysis because experiencing a SARS-CoV-2 outcome during 2020-2021 prior to influenza vaccination may alter the estimates for the YN group (ie, those individuals who could not receive influenza vaccination because they would be censored once they experienced a SARS-CoV-2 outcome).

All statistical tests were 2-tailed, with *P* < .05 defined as the level of statistical significance. We performed our analyses using SAS, version 9.4 software (SAS Institute Inc).

## Results

We identified 2 922 449 individuals aged 66 years or older (54.2% female) living in Ontario at the start of the study period (October 1, 2019). After the exclusions (eTable 2 in the Supplement), 2 279 805 individuals (1 234 647 female [54.2%] and 1 045 158 male [45.8%]) were included in the study. The mean (SD) age of the entire cohort was 75.08 (7.21) years. In the 2019-2020 cohort, 1 229 487 individuals (53.9%) received the influenza vaccine; in the 2020-2021 cohort, 1 266 939 individuals (57.8%) received the vaccine. Table 1 provides the baseline characteristics of the 2 annual cohorts.

**Table 1.** Demographics and Clinical Characteristics of Vaccinated and Unvaccinated Individuals Aged 66 Years or Older (2019-2020 and 2020-2021 Cohorts)

Variable	2019-2020 Cohort: October 1, 2019, to March 31, 2020			2020-2021 Cohort: October 1, 2020, to March 31, 2021		
	No. (%)		Standardized difference^a^	No. (%)		Standardized difference^a^
	Vaccinated (n = 1 229 487)	Unvaccinated (n = 1 050 318)		Vaccinated (n = 1 266 939)	Unvaccinated (n = 924 604)	
Sex						
Female	665 832 (54.2)	568 815 (54.2)	0	688 502 (54.3)	501 276 (54.2)	0
Male	563 655 (45.8)	481 503 (45.8)	0	578 437 (45.7)	423 328 (45.8)	0
Age group, y						
66-74	633 517 (51.5)	623 387 (59.4)	0.16	694 496 (54.8)	540 830 (58.5)	0.07
75-84	432 189 (35.2)	305 609 (29.1)	0.13	433 189 (34.2)	274 341 (29.7)	0.10
≥85	163 781 (13.3)	121 322 (11.6)	0.05	139 254 (11.0)	109 433 (11.8)	0.03
Neighborhood income quintile						
1 (Lowest income)	223 620 (18.2)	219 070 (20.9)	0.07	221 130 (17.5)	199 505 (21.6)	0.10
2	250 343 (20.4)	221 901 (21.1)	0.02	254 868 (20.1)	197 757 (21.4)	0.03
3	246 378 (20.0)	210 851 (20.1)	0	253 404 (20.0)	186 753 (20.2)	0
4	239 743 (19.5)	195 594 (18.6)	0.02	250 315 (19.8)	170 038 (18.4)	0.03
5 (Highest income)	266 851 (21.7)	199 578 (19.0)	0.07	284 576 (22.5)	167 719 (18.1)	0.11
Missing	2552 (0.2)	3324 (0.3)	0.02	2646 (0.2)	2832 (0.3)	0.02
PHU region						
Central East	346 163 (28.2)	275 384 (26.2)	0.04	362 447 (28.6)	234 208 (25.3)	0.07
Central West	53 271 (4.3)	42 477 (4.0)	0.01	54 502 (4.3)	37 560 (4.1)	0.01
Durham	95 495 (7.8)	80 393 (7.7)	0	100 710 (7.9)	68 115 (7.4)	0.02
Eastern	66 098 (5.4)	84 108 (8.0)	0.11	68 780 (5.4)	74 999 (8.1)	0.11
North	90 027 (7.3)	58 794 (5.6)	0.07	92 926 (7.3)	50 323 (5.4)	0.08
Ottawa	92 163 (7.5)	96 507 (9.2)	0.06	93 073 (7.3)	89 429 (9.7)	0.08
Peel	171 847 (14.0)	126 704 (12.1)	0.06	175 955 (13.9)	110 191 (11.9)	0.06
Southwest	219 123 (17.8)	202 276 (19.3)	0.04	220 054 (17.4)	185 058 (20.0)	0.07
Toronto	2196 (0.2)	2956 (0.3)	0.02	2294 (0.2)	2494 (0.3)	0.02
York	93 104 (7.6)	80 719 (7.7)	0	96 198 (7.6)	72 227 (7.8)	0.01
Residence						
Urban	1 084 795 (88.2)	901 711 (85.9)	0.07	1 114 776 (88.0)	794 993 (86.0)	0.06
Rural	142 496 (11.6)	145 651 (13.9)	0.07	149 869 (11.8)	127 117 (13.7)	0.06
Missing	2196 (0.2)	2956 (0.3)	0.02	2294 (0.2)	2494 (0.3)	0.02
Essential worker status^b^						
1 (0%-32.5%)	269 979 (22.0)	200 431 (19.1)	0.07	283 870 (22.4)	170 879 (18.5)	0.10
2 (32.6%-42.3%)	273 956 (22.3)	221 330 (21.1)	0.03	284 504 (22.5)	192 859 (20.9)	0.04
3 (42.4%-49.9%)	250 304 (20.4)	215 580 (20.5)	0	257 705 (20.3)	189 593 (20.5)	0
4 (50.0%-57.5%)	232 920 (18.9)	214 902 (20.5)	0.04	237 696 (18.8)	192 011 (20.8)	0.05
5 (57.6%-114.3%)	197 989 (16.1)	192 688 (18.3)	0.06	198 780 (15.7)	174 566 (18.9)	0.08
Missing	4339 (0.4)	5387 (0.5)	0.02	4384 (0.3)	4696 (0.5)	0.02
Household density quintile^c^						
1 (0-2.1)	298 518 (24.3)	246 216 (23.4)	0.02	304 491 (24.0)	213 850 (23.1)	0.02
2 (2.2-2.4)	243 580 (19.8)	210 691 (20.1)	0.01	251 670 (19.9)	184 122 (19.9)	0
3 (2.5-2.6)	182 953 (14.9)	147 550 (14.0)	0.02	190 050 (15.0)	128 204 (13.9)	0.03
4 (2.7-3.0)	279 565 (22.7)	229 591 (21.9)	0.02	291 105 (23.0)	200 035 (21.6)	0.03
5 (3.1-5.7)	219 956 (17.9)	210 292 (20.0)	0.05	224 742 (17.7)	193 130 (20.9)	0.08
Missing	4915 (0.4)	5978 (0.6)	0.02	4881 (0.4)	5263 (0.6)	0.03
Limited educational attainment quintile^d^						
1 (0.0%-4.1%)	269 123 (21.9)	197 358 (18.8)	0.08	283 166 (22.4)	167 531 (18.1)	0.11
2 (4.2%-7.5%)	274 891 (22.4)	216 859 (20.6)	0.04	286 454 (22.6)	187 317 (20.3)	0.06
3 (7.6%-11.4%)	255 503 (20.8)	219 516 (20.9)	0	263 721 (20.8)	193 442 (20.9)	0
4 (11.5%-17.1%)	235 515 (19.2)	218 542 (20.8)	0.04	239 366 (18.9)	195 751 (21.2)	0.06
5 (17.2%-94.3%)	190 136 (15.5)	192 673 (18.3)	0.08	189 866 (15.0)	175 883 (19.0)	0.11
Missing	4319 (0.4)	5370 (0.5)	0.02	4366 (0.3)	4680 (0.5)	0.02
Uncoupled quintile^e^						
1 (11.2%-33.7%)	280 219 (22.8)	226 979 (21.6)	0.03	296 594 (23.4)	195 485 (21.1)	0.05
2 (33.8%-38.4%)	236 904 (19.3)	195 955 (18.7)	0.02	248 430 (19.6)	170 884 (18.5)	0.03
3 (38.5%-43.6%)	231 705 (18.8)	194 667 (18.5)	0.01	238 743 (18.8)	172 090 (18.6)	0.01
4 (43.7%-51.0%)	245 607 (20.0)	210 111 (20.0)	0	249 243 (19.7)	187 147 (20.2)	0.01
5 (51.1%-94.6%)	230 137 (18.7)	216 628 (20.6)	0.05	229 048 (18.1)	193 735 (21.0)	0.07
Missing	4915 (0.4)	5978 (0.6)	0.02	4881 (0.4)	5263 (0.6)	0.03
Self-identify as visible minority quintile^f^						
1 (0.0%-2.2%)	230 428 (18.7)	210 236 (20.0)	0.03	240 341 (19.0)	182 825 (19.8)	0.02
2 (2.3%-7.5%)	252 283 (20.5)	198 865 (18.9)	0.04	263 558 (20.8)	169 271 (18.3)	0.06
3 (7.6%-18.7%)	252 250 (20.5)	187 883 (17.9)	0.07	262 980 (20.8)	159 646 (17.3)	0.09
4 (18.8%-43.5%)	239 059 (19.4)	202 565 (19.3)	0	247 114 (19.5)	177 290 (19.2)	0.01
5 (43.6%-102%)	251 186 (20.4)	245 414 (23.4)	0.07	248 607 (19.6)	230 915 (25.0)	0.13
Missing	4281 (0.3)	5355 (0.5)	0.02	4339 (0.3)	4657 (0.5)	0.02
Immigrated last 5 y^g^						
1 (0.0%-2.1%)	728 000 (59.2)	595 892 (56.7)	0.05	758 402 (59.9)	513 353 (55.5)	0.09
2 (2.2%-4.7%)	235 930 (19.2)	197 964 (18.8)	0.01	242 573 (19.1)	174 743 (18.9)	0.01
3 (4.8%-41.2%)	259 175 (21.1)	246 609 (23.5)	0.06	259 576 (20.5)	227 629 (24.6)	0.10
Missing	6382 (0.5)	9853 (0.9)	0.05	6388 (0.5)	8879 (1.0)	0.05
Apartment building density category^h^						
1 (0%-7.3%)	683 913 (55.6)	573 008 (54.6)	0.02	712 296 (56.2)	503 119 (54.4)	0.04
2 (7.4%-37.7%)	230 767 (18.8)	200 887 (19.1)	0.01	237 025 (18.7)	176 087 (19.0)	0.01
3 (37.8%-104%)	309 864 (25.2)	270 418 (25.7)	0.01	312 706 (24.7)	240 112 (26.0)	0.03
Missing	4943 (0.4)	6005 (0.6)	0.02	4912 (0.4)	5286 (0.6)	0.03
Any comorbidity	1 060 809 (86.3)	829 824 (79.0)	0.19	1 075 199 (84.9)	730 708 (79.0)	0.15
Asthma	187 001 (15.2)	124 206 (11.8)	0.10	187 713 (14.8)	109 249 (11.8)	0.09
COPD	266 369 (21.7)	198 700 (18.9)	0.07	261 434 (20.6)	172 097 (18.6)	0.05
Congestive heart failure	111 672 (9.1)	86 895 (8.3)	0.03	99 895 (7.9)	73 153 (7.9)	0
Ischemic heart disease	133 196 (10.8)	101 499 (9.7)	0.04	130 308 (10.3)	87 585 (9.5)	0.03
Hypertension	896 702 (72.9)	672 184 (64.0)	0.19	902 875 (71.3)	592 990 (64.1)	0.15
Diabetes	393 441 (32.0)	297 430 (28.3)	0.08	388 601 (30.7)	266 886 (28.9)	0.04
HIV infected	1156 (0.1)	812 (0.1)	0.01	1191 (0.1)	686 (0.1)	0.01
Solid organ or stem cell transplant	3607 (0.3)	2175 (0.2)	0.02	3464 (0.3)	1796 (0.2)	0.02
Other immune system disorders	20 917 (1.7)	15 679 (1.5)	0.02	21 402 (1.7)	13 438 (1.5)	0.02
Immunosuppressive therapy	75 949 (6.2)	49 654 (4.7)	0.06	74 130 (5.9)	40 850 (4.4)	0.06
Active cancer	37 367 (3.0)	29 770 (2.8)	0.01	35 229 (2.8)	21 207 (2.3)	0.03
Autoimmune disorders	42 097 (3.4)	29 057 (2.8)	0.04	42 461 (3.4)	24 960 (2.7)	0.04
Rheumatoid arthritis	36 495 (3.0)	25 392 (2.4)	0.03	36 651 (2.9)	21 905 (2.4)	0.03
Inflammatory bowel disease	5953 (0.5)	3864 (0.4)	0.02	6164 (0.5)	3223 (0.3)	0.02
Chronic kidney disease or long-term dialysis (≥3 consecutive months)	105 057 (8.5)	80 039 (7.6)	0.03	96 662 (7.6)	69 131 (7.5)	0.01
Long-term dialysis	2009 (0.2)	4034 (0.4)	0.04	1364 (0.1)	3269 (0.4)	0.05
Chronic kidney disease	105 055 (8.5)	80 039 (7.6)	0.03	96 660 (7.6)	69 131 (7.5)	0.01
Advanced liver disease	17 857 (1.5)	14 580 (1.4)	0.01	17 194 (1.4)	12 087 (1.3)	0
Cirrhosis	17 203 (1.4)	13 847 (1.3)	0.01	16 595 (1.3)	11 592 (1.3)	0
Decompensated cirrhosis	1841 (0.1)	2043 (0.2)	0.01	1623 (0.1)	1435 (0.2)	0.01
Dementia	61 850 (5.0)	57 973 (5.5)	0.02	49 613 (3.9)	47 216 (5.1)	0.06
Frailty	14 844 (1.2)	19 423 (1.8)	0.05	10 833 (0.9)	13 573 (1.5)	0.06
History of stroke or transient ischemic attack	57 319 (4.7)	49 182 (4.7)	0	53 609 (4.2)	42 442 (4.6)	0.02
No. of acute-care hospital admissions in past 3 y						
0	934 171 (76.0)	813 413 (77.4)	0.03	982 717 (77.6)	725 334 (78.4)	0.02
1	195 901 (15.9)	150 800 (14.4)	0.04	194 135 (15.3)	130 660 (14.1)	0.03
2	60 841 (4.9)	49 147 (4.7)	0.01	57 453 (4.5)	40 694 (4.4)	0.01
≥3	38 574 (3.1)	36 958 (3.5)	0.02	32 634 (2.6)	27 916 (3.0)	0.03
No. of physician office visits in past 1 y						
0	36 100 (2.9)	130 268 (12.4)	0.36	42 131 (3.3)	119 829 (13.0)	0.36
1	55 544 (4.5)	83 907 (8.0)	0.14	60 875 (4.8)	74 702 (8.1)	0.13
2-4	260 739 (21.2)	261 223 (24.9)	0.09	275 516 (21.7)	232 225 (25.1)	0.08
5-8	350 252 (28.5)	256 466 (24.4)	0.09	361 285 (28.5)	226 637 (24.5)	0.09
9-14	305 423 (24.8)	189 985 (18.1)	0.17	309 866 (24.5)	165 153 (17.9)	0.16
≥15	221 429 (18.0)	128 469 (12.2)	0.16	217 266 (17.1)	106 058 (11.5)	0.16

Abbreviations: COPD, chronic obstructive pulmonary disease; PHU, public health unit.

^a^ Standardized differences greater than 0.10 are considered clinically relevant.

^b^ Parenthetical ranges indicate the percentage of individuals in the area working in the following occupations: sales and service occupations; trades, transport and equipment operators, and related occupations; natural resources, agriculture, and related production occupations; and occupations in manufacturing and utilities. Census counts for people are randomly rounded up or down to the nearest number divisible by 5, which causes some minor imprecision.

^c^ Parenthetical ranges represent the range of persons per dwelling.

^d^ Parenthetical ranges represent percentage of adult individuals aged 25 to 64 years in the area who have not received any type of diploma.

^e^ Individuals who have never legally married and are not living with a person as a couple; separated (people who are married but who are no longer living with their spouse [for reasons other than, for example, illness, work, or school], have not obtained a divorce and are not living with a person as a couple); divorced (people who have obtained a legal divorce, have not remarried, and are not living with a person as a couple); and widowed (people who have lost their married spouse through death, have not remarried, and are not living with a person as a couple).

^f^ Quintile representing individuals in the area self-identifying as a visible minority.

^g^ Parenthetical ranges represent percentage of individuals in the area who are recent immigrants.

^h^ Census counts are randomly rounded up or down to the nearest number divisible by 5, which causes some minor imprecision: 7.3% represents the 60th percentile.

### Association of Influenza Vaccination With SARS-CoV-2 Infection and Associated Hospitalization and Mortality

For the 2019-2020 cohort, influenza vaccination was negatively associated with SARS-CoV-2 infection (aHR, 0.78; 95% CI, 0.73-0.84) (Table 2). We observed a similar negative association for the 2020-2021 cohort (aHR, 0.76; 95% CI, 0.74-0.78). In addition, influenza vaccination was inversely associated with the risk of SARS-CoV-2–associated hospitalization in the 2019-2020 cohort (aHR, 0.83; 95% CI, 0.75-0.93) and even more so in the 2020-2021 cohort (aHR, 0.68; 95% CI, 0.64-0.72). The risk of SARS-CoV-2–associated mortality was also lower for individuals who received the influenza vaccine in the 2019-2020 cohort (aHR, 0.74; 95% CI, 0.62-0.87) and 2020-2021 cohort (aHR, 0.58; 95% CI, 0.53-0.63). The results were similar for the association between influenza vaccination and the composite outcome of SARS-CoV-2–associated hospitalization/mortality (2019-2020 cohort: aHR, 0.83; 95% CI, 0.74-0.92; 2020-2021 cohort: aHR, 0.66; 95% CI, 0.63-0.70).

**Table 2.** Association Between Receipt of Influenza Vaccines in the 2019-2020 and 2020-2021 Cohorts, PHE in 2018-2019, and Study Outcomes

	2019-2020 Cohort		2020-2021 Cohort		PHE, 2018-2019^a^	
	Vaccinated	Unvaccinated	Vaccinated	Unvaccinated	Yes	No
No. of individuals (%)	1 229 487 (53.9)	1 050 318 (46.1)	1 266 939 (57.8)	924 604 (42.2)	342 800 (15.0)	1 937 005 (85.0)
SARS-CoV-2 infection						
No. of events (%)	2584 (0.21)	2745 (0.26)	13 188 (1.04)	15 227 (1.65)	748 (0.22)	4582 (0.24)
Unadjusted HR (95% CI)	0.79 (0.75-0.83)	1 [Reference]	0.73 (0.71-0.75)	1 [Reference]	0.91 (0.84-0.98)	1 [Reference]
Age-sex adjusted HR (95% CI)	0.76 (0.72-0.80)	1 [Reference]	0.73 (0.71-0.75)	1 [Reference]	0.95 (0.88-1.03)	1 [Reference]
Fully adjusted HR (95% CI)^b^	0.78 (0.73-0.84)	1 [Reference]	0.76 (0.74-0.78)	1 [Reference]	0.85 (0.78-0.91)	1 [Reference]
SARS-CoV-2–associated hospitalization						
No. of events (%)	992 (0.08)	980 (0.09)	3508 (0.28)	4704 (0.51)	273 (0.08)	1939 (0.10)
Unadjusted HR (95% CI)	0.84 (0.77-0.92)	1 [Reference]	0.68 (0.65-0.71)	1 [Reference]	0.79 (0.70-0.90)	1 [Reference]
Age-sex adjusted HR (95% CI)	0.78 (0.72-0.86)	1 [Reference]	0.68 (0.65-0.71)	1 [Reference]	0.85 (0.75-0.97)	1 [Reference]
Fully adjusted HR (95% CI)^b^	0.83 (0.75-0.93)	1 [Reference]	0.68 (0.64-0.72)	1 [Reference]	0.79 (0.69-0.90)	1 [Reference]
SARS-CoV-2–associated death						
No. of events (%)	428 (0.03)	441 (0.04)	1210 (0.10)	1862 (0.20)	106 (0.03)	791 (0.04)
Unadjusted HR (95% CI)	0.82 (0.72-0.93)	1 [Reference]	0.60 (0.56-0.65)	1 [Reference]	0.77 (0.63-0.94)	1 [Reference]
Age-sex adjusted HR (95% CI)	0.73 (0.64-0.84)	1 [Reference]	0.60 (0.55-0.64)	1 [Reference]	0.88 (0.72-1.08)	1 [Reference]
Fully adjusted HR (95% CI)^b^	0.74 (0.62-0.87)	1 [Reference]	0.58 (0.53-0.63)	1 [Reference]	0.82 (0.67-1.01)	1 [Reference]
SARS-CoV-2–associated hospitalization or death						
No. of events (%)	1104 (0.09)	1093 (0.10)	3788 (0.30)	5160 (0.56)	289 (0.08)	2060 (0.11)
Unadjusted HR (95% CI)	0.84 (0.77-0.91)	1 [Reference]	0.66 (0.64-0.69)	1 [Reference]	0.79 (0.70-0.90)	1 [Reference]
Age-sex adjusted HR (95% CI)	0.78 (0.71-0.85)	1 [Reference]	0.66 (0.64-0.69)	1 [Reference]	0.86 (0.76-0.97)	1 [Reference]
Fully adjusted HR (95% CI)^b^	0.83 (0.74-0.92)	1 [Reference]	0.66 (0.63-0.70)	1 [Reference]	0.80 (0.70-0.90)	1 [Reference]
SARS-CoV-2 testing						
No. of events (%)	209 077 (17.0)	154 845 (14.7)	254 093 (20.1)	183 217 (19.8)	48 986 (14.3)	314 937 (16.3)
Unadjusted OR (95% CI)	1.19 (1.18-1.19)	1 [Reference]	1.02 (1.01-1.02)	1 [Reference]	0.86 (0.85-0.87)	1 [Reference]
Age-sex adjusted OR (95% CI)	1.17 (1.16-1.17)	1 [Reference]	1.02 (1.01-1.03)	1 [Reference]	0.89 (0.88-0.90)	1 [Reference]
Fully adjusted OR (95% CI)^b^	1.05 (1.04-1.06)	1 [Reference]	0.91 (0.90-0.92)	1 [Reference]	0.91 (0.90-0.92)	1 [Reference]

Abbreviations: HR, hazard ratio; OR, odds ratio; PHE, periodic health examination.

^a^ We assessed the association between PHE and COVID-19 outcomes in the 2019-2020 cohort.

^b^ Adjusted for age, sex, comorbidities, past health care utilization, receipt of previous-year influenza vaccination, PHE (past year), neighborhood income quintile, rurality, public health unit region, and census variables.

### Likelihood of SARS-CoV-2 Testing

For the 2019-2020 cohort, the frequency of SARS-CoV-2 testing was significantly higher in influenza-vaccinated individuals (AOR, 1.05; 95% CI, 1.04-1.06). For the 2020-2021 cohort, we observed a lower frequency of SARS-CoV-2 testing in influenza-vaccinated individuals (AOR, 0.91; 95% CI, 0.90-0.92) (Table 2).

### PHE and SARS-CoV-2 Outcomes

We examined the association between PHE in 2018-2019 and SARS-CoV-2 infection in the 2019-2020 cohort. A total of 342 800 individuals (15.0%) in this annual cohort underwent a PHE, and undergoing a PHE was associated with a lower risk of SARS-CoV-2 infection in the next year (aHR of SARS-CoV-2 infection, 0.85; 95% CI, 0.78-0.91) (Table 2). Similarly, we observed a significant negative association between PHE and SARS-CoV-2–associated hospitalization (aHR, 0.79; 95% CI, 0.69-0.90). Undergoing a PHE was also marginally associated with a lower risk of SARS-CoV-2–associated mortality (aHR, 0.82; 95% CI, 0.67-1.01). In addition, undergoing a PHE was also associated with a lower incidence of SARS-CoV-2–associated hospitalization or death (aHR, 0.80; 95% CI, 0.70-0.90). People who underwent a PHE were less likely to be tested for COVID-19 (14.3% vs 16.3%; AOR, 0.91; 95% CI, 0.90-0.92).

### Influenza Vaccination and PHE

Individuals who underwent a PHE in 2018-2019 were more likely to have received an influenza vaccine in the 2019-2020 cohort (209 563 of 342 800 [61.1%]) compared with those who did not (993 990 of 1 937 005 [51.3%]; relative risk, 1.19; 95% CI, 1.19-1.20). Owing to the significant association between PHE and influenza vaccination, we stratified our analysis of influenza vaccines and the risk of study outcomes by PHE. We observed that the aHR for the association between influenza vaccination and SARS-CoV-2 infection (aHR, 0.78; 95% CI, 0.73-0.84) was considerably decreased for vaccinated individuals who underwent a PHE (aHR, 0.62; 95% CI, 0.52-0.74) than for those who did not undergo PHE (aHR, 0.81; 95% CI, 0.76-0.87) (Table 3). This difference was also observed for SARS-CoV-2–associated hospitalization (aHR for vaccinated individuals with PHE, 0.64; 95% CI, 0.47-0.87; aHR for vaccinated individuals without PHE, 0.86; 95% CI, 0.77-0.97), SARS-CoV-2–associated mortality (aHR for vaccinated individuals with PHE, 0.46; 95% CI, 0.28-0.74; aHR for vaccinated individuals without PHE, 0.78; 95% CI, 0.66-0.92), and SARS-CoV-2–associated hospitalization/mortality (aHR for vaccinated individuals with PHE, 0.66; 95% CI, 0.49-0.88; aHR for vaccinated individuals without PHE, 0.85; 95% CI, 0.76-0.95).

**Table 3.** Classification of the Association Between Influenza Vaccination and COVID-19 Outcomes Based on Receipt of PHE

	2019-2020 Cohort	
	Vaccinated	Unvaccinated
No. of individuals (%)	1 229 487 (53.9)	1 050 318 (46.1)
SARS-CoV-2 infection		
No. of events (%)	2584 (0.21)	2745 (0.26)
HR (95% CI)	0.79 (0.75-0.83)	1 [Reference]
PHE 2018-2019, fully adjusted HR (95% CI)		
Yes	0.62 (0.52-0.74)	
No	0.81 (0.76-0.87)	
SARS-CoV-2–associated hospitalization		
No. of events (%)	992 (0.08)	980 (0.09)
HR (95% CI)	0.84 (0.77-0.92)	1 [Reference]
PHE 2018-2019, fully adjusted HR (95% CI)		
Yes	0.64 (0.47-0.87)	
No	0.86 (0.77-0.97)	
SARS-CoV-2–associated death		
No. of events (%)	428 (0.03)	441 (0.04)
HR (95% CI)	0.82 (0.72-0.93)	1 [Reference]
PHE 2018-2019, fully adjusted HR (95% CI)		
Yes	0.46 (0.28-0.74)	
No	0.78 (0.66-0.92)	
SARS-CoV-2–associated hospitalization or death		
No. of events (%)	1104 (0.09)	1093 (0.10)
HR (95% CI)	0.84 (0.77-0.91)	1 [Reference]
PHE 2018-2019, fully adjusted HR (95% CI)		
Yes	0.66 (0.49-0.88)	
No	0.85 (0.76-0.95)	

Abbreviations: HR, hazard ratio; PHE, periodic health examination.

### Repeated Influenza Vaccination and SARS-CoV-2 Outcomes

The YY group had a significantly lower incidence of SARS-CoV-2 infection than that of the NN group (aHR, 0.67; 95% CI, 0.63-0.72) (Table 4). The NY group also had a lower incidence of SARS-CoV-2 infection than the NN group (aHR, 0.91; 95% CI, 0.83-0.99) had, but the YN group did not (aHR, 1.00; 95% CI, 0.92-1.10).

**Table 4.** Association Between Influenza Vaccination in 2018 and 2019 on the SARS-CoV-2 Outcomes in the 2019-2020 Cohort, Fully-Adjusted Model

2019-2020 Cohort^a^	aHR (95% CI)
SARS-CoV-2 infection	
Influenza vaccination	
NN	1 [Reference]
YN	1.00 (0.92-1.10)
NY	0.91 (0.83-0.99)
YY	0.67 (0.63-0.72)
SARS-CoV-2–associated hospitalization	
Influenza vaccination	
NN	1 [Reference]
YN	0.94 (0.81-1.10)
NY	0.92 (0.79-1.07)
YY	0.70 (0.63-0.78)
SARS-CoV-2–associated death	
Influenza vaccination	
NN	1 [Reference]
YN	1.06 (0.85-1.31)
NY	0.86 (0.69-1.09)
YY	0.68 (0.58-0.79)
SARS-CoV-2–associated hospitalization or death	
Influenza vaccination	
NN	1 [Reference]
YN	0.95 (0.82-1.09)
NY	0.91 (0.79-1.05)
YY	0.70 (0.63-0.77)

Abbreviation: aHR, adjusted hazard ratio.

^a^ We considered individuals who did not receive influenza vaccination in either 2018-2019 or 2019-2020 as the reference group (NN). YN indicates individuals who received influenza vaccination in 2018-2019 but not in 2019-2020; NY, individuals who did not receive influenza vaccination in 2018-2019 but received it in 2019-2020; and YY, Individuals who received influenza vaccination in both 2018-2019 and 2019-2020.

Similarly, the YY group had a significantly lower incidence of SARS-CoV-2 hospitalization/death compared with the NN group (aHR, 0.70; 95% CI, 0.63-0.77). The association between influenza vaccination and SARS-CoV-2 hospitalization/death was not statistically significant for the NY and YN groups (aHR, 0.91; 95% CI, 0.79-1.05 and aHR, 0.95; 95% CI, 0.82-1.09), respectively.

## Discussion

We demonstrated that influenza vaccination during the 2019-2020 and 2020-2021 vaccination campaigns was associated with a 22% to 24% lower risk of SARS-CoV-2 infection, a 17% to 32% lower risk of SARS-CoV-2–associated hospitalization, and a 27% to 42% lower risk of SARS-CoV-2–associated mortality during the first 2 years of the COVID-19 pandemic. However, we also found that undergoing a PHE during 2018-2019 was associated with a 15% lower risk of SARS-CoV-2 infection, a 21% lower risk of SARS-CoV-2–associated hospitalization, and an 18% lower risk of SARS-CoV-2–associated mortality during 2019-2020. We subsequently showed that the negative association between influenza vaccination and SARS-CoV-2–related outcomes differed considerably when the analysis was stratified by a history of PHE in the preceding year. This finding suggests that the negative association between influenza vaccination and SARS-CoV-2–related outcomes was at least partially overestimated because of the healthy vaccinee bias. In addition, we showed that aHRs for the associations were decreased for individuals who had received the influenza vaccine during 2 consecutive seasons than for those who had received it during the current season only, which further suggests the presence of healthy vaccinee bias.

Observational studies are susceptible to potential sources of bias associated with selection of the study population, misclassification, and confounded measurements. In this large-scale, population-based study, we showed the importance of considering the simultaneous effects of variables, which may alter the estimation of vaccine effectiveness. The frequency of SARS-CoV-2 testing was greater in influenza-vaccinated vs unvaccinated individuals for the 2019-2020 cohort, while this association was negative for the 2020-2021 cohort.

Other observational studies have reported a negative association between influenza vaccination and SARS-CoV-2–related outcomes.^3,5,17^ This negative association was also demonstrated by estimating the odds of testing positive for SARS-CoV-2 in patients who received the influenza vaccine compared with those who did not.^9,18^ Although these observational studies showed that individuals who received influenza vaccines were at reduced risk of experiencing SARS-CoV-2–related outcomes, potential sources of healthy vaccinee bias have not been investigated.^19^

The protective effect of influenza vaccination against SARS-CoV-2 infection and severe outcomes would appear implausible. Some studies hypothesized that “trained immunity” following influenza vaccination may alter the SARS-CoV-2 infection risk.^18,20,21^ Experimental studies have suggested that influenza vaccines may provide a temporary preventive effect against noninfluenza respiratory infections by inducing innate immunity through various mechanisms.^22,23^ The expression of interferon genes was found to be induced by influenza vaccines immediately after vaccination.^24^ In addition, a study has hypothesized that exposure to the influenza virus confers short-term immunity against other respiratory viruses, at least for a few weeks, because of the activation of a nonspecific immune response mediated by the release of interferons and other cytokines.^10^ Although this phenomenon was suggested to provide a protective effect against several respiratory viruses,^25^ this protection should only be transient.^22^ Thus, the protective effect of influenza vaccination against infection with noninfluenza respiratory viruses does not appear biologically plausible. Moreover, this “heterologous immunity” against other viruses may cause harm (eg, autoimmune disorders) rather than benefits.^26^

Nelson et al^14^ explained the likelihood of influenza vaccine effectiveness overestimation during influenza seasons secondary to healthy vaccinee bias. According to this concept, individuals with health-promoting behaviors are more likely to adhere to the annually recommended vaccination. Subsequently, Remschmidt et al^12^ showed that the healthy vaccinee bias could have influenced multiple influenza vaccine effectiveness studies. To explore this bias, we initially calculated the HRs of SARS-CoV-2–related outcomes adjusted for comorbidities and other potential confounders. The estimated aHRs showed that this strategy might not overcome the risk of healthy vaccinee bias, as the negative association between influenza vaccination and SARS-CoV-2–related outcomes remained statistically significant, with fully adjusted HRs. In some epidemiologic studies that assessed the effectiveness of influenza vaccines in older adults, the individuals who were classified as “healthy” because they lacked diagnostic codes for comorbidities (eg, diabetes, cancer, and heart, lung, and kidney disorders) were more likely to be cognitively or functionally impaired based on hospital medical record reviews.^27^ Hence, although healthy adherers may truly have a lower rate of comorbidities, hazard estimates adjusted for comorbidities recorded in health administrative databases may not successfully eliminate the risk of healthy vaccinee bias.

Of note, the term *healthy* in this bias refers to health consciousness and not necessarily a lack of comorbidities. The healthy vaccinee bias may be more conspicuous for influenza vaccination compared with other immunizations.^14^ Although individuals with health-promoting behavior are more likely to receive the influenza vaccine, reduced adherence to vaccination by the individuals with deteriorating health status may lead to overestimating mortality risk reduction.^28^

Receiving the influenza vaccine is voluntary, and individuals who are more cautious about their health are more likely to receive vaccines. Regardless of their comorbidities, these individuals are likely more motivated and inclined to engage in health-promoting and disease-preventing behaviors. Therefore, to overcome the healthy vaccinee bias, vaccine effectiveness studies should consider some strategies to identify these individuals.

The study population in SARS-CoV-2 vaccine trials is typically restricted to healthy volunteers, and their well-defined end points are usually measured under ideal settings.^1,29^ Thus, population-based studies are needed to estimate SARS-CoV-2 vaccine effectiveness in real-world conditions.^30^ The exact influence of the vaccines cannot be estimated unless potential sources of bias, such as the healthy vaccinee effect, are corrected. Most vaccine effectiveness studies did not implement specific strategies to reduce the bias arising from differences in health care–seeking behavior between vaccinated and unvaccinated individuals.^31,32,33,34,35,36^ Some studies measured the weight of an observation by the inverse of the propensity to be vaccinated.^31^ However, none of these strategies specifically targeted the healthy vaccinee effect to reduce the risk of bias. Although multivariable adjustment for demographic variables and comorbidities likely decreases the risk of confounding bias, the risk of healthy vaccinee bias may not be eliminated.^37,38^ Studies can implement different strategies to detect potential proxies of the healthy vaccinee bias, such as frequent health care system contact to monitor health status, insurance condition, functional limitations, living alone, awareness of vaccine recommendations by health authorities, and mobilization status.^39,40,41^

### Limitations

This study has limitations. First, we were not able to identify undiagnosed patients who were not tested for SARS-CoV-2. Second, 14-day hospitalization and 30-day mortality after a positive SARS-CoV-2 test result are all-cause outcomes, and individuals may have been admitted to hospitals or died for reasons unrelated to SARS-CoV-2 infection. Third, measurement of influenza vaccination using physician and pharmacist billing claims has imperfect sensitivity and specificity; thus, some misclassification of the exposure variable may have occurred.^42^ Fourth, as with all observational studies, unmeasured confounding may persist.

## Conclusions

In this cohort study, we observed a lower risk of SARS-CoV-2 infection and associated hospitalization and mortality in older adults who received influenza vaccination in 2 provincial, annual cohorts. We found a similar negative association between undertaking a PHE in the preceding year and occurrence of SARS-CoV-2–related outcomes. The association between influenza vaccine and SARS-CoV-2 outcomes was at least partially modified by whether an individual had undergone a PHE, suggesting that PHE may be a proxy for the presence of healthy vaccinee bias. Our findings may have implications for other influenza vaccine effectiveness studies in adults aged 66 years or older.

## Notes

Supplement. **eTable 1.** Checklist of Reporting of Studies Conducted Using Observational Routinely-Collected Health Data (RECORD) Statement **eTable 2.** Number of Individuals With Inclusion and Exclusion Criteria **eTable 3.** Variable Definitions
eReferences
