# Supplementary material for: Association of Influenza Vaccination With SARS-CoV-2 Infection and Associated Hospitalization and Mortality Among Patients Aged 66 Years or Older
Source: JAMA Netw Open. 2022 Sep 28;5(9):e2233730. doi: 10.1001/jamanetworkopen.2022.33730 (PMC9520345; doi:10.1001/jamanetworkopen.2022.33730)
Supplement: Supplement. — eTable 1. Checklist of Reporting of Studies Conducted Using Observational Routinely-Collected Health Data (RECORD) Statement eTable 2. Number of Individuals With Inclusion and Exclusion Criteria eTable 3. Variable Definitions eReferences [file jamanetwopen-e2233730-s001.pdf]

Supplementary Online Content

Hosseini-Moghaddam SM, He S, Calzavara A, Campitelli MA, Kwong JC. Association of influenza vaccination with SARS-CoV-2 infection and associated hospitalization and mortality among patients aged 66 years or older. *JAMA Netw Open.* 2022;5(9):e2233730. doi:10.1001/jamanetworkopen.2022.33730

eTable 1. Checklist of Reporting of Studies Conducted Using Observational Routinely-Collected Health Data (RECORD) Statement

eTable 2. Number of Individuals With Inclusion and Exclusion Criteria

eTable 3. Variable Definitions

eReferences

This supplementary material has been provided by the authors to give readers additional information about their work.

© 2022 Hosseini-Moghaddam SM et al. *JAMA Network Open.*

eTable 1. Checklist of Reporting of Studies Conducted Using Observational Routinely-Collected Health Data (RECORD) Statement

	Item No	STROBE items	RECORD items	Reported
Title and abstract	1	(a) Indicate the study's design with a commonly used term in the title or the abstract.<br>(b) Provide in the abstract an informative and balanced summary of what was done and what was found.	(1.1) The type of data used should be specified in the title or abstract. When possible, the name of the databases used should be included.<br>(1.2) If applicable, the geographic region and time frame within which the study took place should be reported in the title or abstract.<br>(1.3) If linkage between databases was conducted for the study, this should be clearly stated in the title or abstract.	Abstract
Introduction				
Background/ rationale	2	Explain the scientific background and rationale for the investigation being reported.		Introduction
Objectives	3	State specific objectives, including any prespecified hypotheses.		Introduction
Methods				
Study design	4	Present key elements of study design early in the paper.		Methods
Setting	5	Describe the setting, locations, and relevant dates, including periods of recruitment, exposure, follow-up, and data collection.		Methods
Participants	6	(a) Give the eligibility criteria, and the sources and methods of selection of participants. Describe methods of follow-up.<br>(b) For matched studies, give matching criteria and number of exposed and unexposed.	(6.1) The methods of study population selection (such as codes or algorithms used to identify subjects) should be listed in detail. If this is not possible, an explanation should be provided.<br>(6.2) Any validation studies of the codes or algorithms used to select the population should be referenced. If validation was conducted for this study and not published elsewhere, detailed methods and results should be provided.<br>(6.3) If the study involved linkage of databases, consider use of a flow diagram or other graphical display to demonstrate the data linkage process, including the number of individuals with linked data at each stage.	Methods, Appendix
Variables	7	Clearly define all outcomes, exposures, predictors, potential confounders, and effect modifiers. Give diagnostic criteria, if applicable.	(7.1) A complete list of codes and algorithms used to classify exposures, outcomes, confounders, and effect modifiers should be provided. If these cannot be reported, an explanation should be provided.	Methods, Appendix

© 2022 Hosseini-Moghaddam SM et al. *JAMA Network Open.*

	Item No	STROBE items	RECORD items	Reported
Data sources/ measurement	8	For each variable of interest, give sources of data and details of methods of assessment (measurement). Describe comparability of assessment methods if there is more than one group.		Methods, Appendix
Bias	9	Describe any efforts to address potential sources of bias.		Methods, Discussion
Study size	10	Explain how the study size was arrived at.		Methods, Results
Quantitative variables	11	Explain how quantitative variables were handled in the analyses. If applicable, describe which groupings were chosen and why.		Methods
Statistical methods	12	(a) Describe all statistical methods, including those used to control for confounding.<br>(b) Describe any methods used to examine subgroups and interactions.<br>(c) Explain how missing data were addressed.<br>(d) If applicable, explain how loss to follow-up was addressed.<br>(e) Describe any sensitivity analyses.		Methods
Data access and cleaning methods		N/A	(12.1) Authors should describe the extent to which the investigators had access to the database population used to create the study population.<br>(12.2) Authors should provide information on the data cleaning methods used in the study.	Methods
Linkage		N/A	(12.3) State whether the study included person-level, institutional-level, or other data linkage across two or more databases. The methods of linkage and methods of linkage quality evaluation should be provided.	Methods
Results				
Participants	13	(a) Report numbers of individuals at each stage of study--e.g. numbers potentially eligible, examined for eligibility, confirmed eligible, included in the study, completing follow-up, and analyzed.<br>(b) Give reasons for non-participation at each stage.<br>(c) Consider use of a flow diagram.	(13.1) Describe in detail the selection of the persons included in the study (i.e., study population selection), including filtering based on data quality, data availability, and linkage. The selection of included persons can be described in the text and/or by means of the study flow diagram.	Methods, Results
Descriptive data	14	(a) Give characteristics of study participants (e.g. demographic, clinical, social) and information on exposures and potential confounders.<br>(b) Indicate number of participants with missing data for each variable of interest.<br>(c) Summarize follow-up time (e.g. average and total amount).		Results
Outcome data	15	Report numbers of outcome events or summary measures over time.		Results

© 2022 Hosseini-Moghaddam SM et al. *JAMA Network Open.*

	Item No	STROBE items	RECORD items	Reported
Main results	16	(a) Give unadjusted estimates and, if applicable, confounder-adjusted estimates and their precision (e.g. 95% confidence interval). Make clear which confounders were adjusted for and why they were included.<br>(b) Report category boundaries when continuous variables were categorized.<br>(c) If relevant, consider translating estimates of relative risk into absolute risk for a meaningful time period.		Results
Other analyses	17	Report other analyses done (e.g. analyses of subgroups and interactions, and sensitivity analyses).		Results
Key results	18	Summarize key results with reference to study objectives.		Discussion
Limitations	19	Discuss limitations of the study, taking into account sources of potential bias or imprecision. Discuss both direction and magnitude of any potential bias.	(19.1) Discuss the implications of using data that were not created or collected to answer the specific research question(s). Include discussion of misclassification bias, unmeasured confounding, missing data, and changing eligibility over time, as they pertain to the study being reported.	Discussion
Interpretation	20	Give a cautious overall interpretation of results considering objectives, limitations, multiplicity of analyses, results from similar studies, and other relevant evidence.		Discussion
Generalizability	21	Discuss the generalizability (external validity) of the study results.		Discussion
Other information				
Funding	22	Give the source of funding and the role of the funders for the present study and, if applicable, for the original study on which the present article is based.		Acknowledgments & Funding
Accessibility of protocol, raw data, and programming code		N/A	(22.1) Authors should provide information on how to access any supplemental information such as the study protocol, raw data, or programming code.	Methods, Appendix

© 2022 Hosseini-Moghaddam SM et al. *JAMA Network Open.*

eTable 2. Number of Individuals With Inclusion and Exclusion Criteria

	N (%)	Cumulative Frequency
Included (age ≥66 years)	2,922,449	2,922,449
Excluded		
Missing birth date or sex	0 (0.00)	2,922,449
Not an Ontario resident	154,899 (5.30)	2,767,550
Not eligible for the Ontario Health Insurance Plan (OHIP)	276,154 (9.45)	2,491,396
No contact with the health care system in the past 3 years	137,085 (4.69)	2,354,311
Age > 105 years at cohort entry	143 (0.01)	2,354,168
Long-Term Care resident	74,363 (2.54)	2,279,805

© 2022 Hosseini-Moghaddam SM et al. *JAMA Network Open.*

eTable 3. Variable Definitions

Variable	Definition
Age	For individuals tested in OLIS, we determined age from their test records in OLIS. For individuals not tested in OLIS, we determined age from the Registered Persons Database. This variable was included *a priori* as considered as a risk factor for COVID-19. [1]
Sex	For individuals tested in OLIS, we determined sex from their test records in OLIS. For individuals not tested in OLIS, we determined sex from the Registered Persons Database. This variable was included *a priori* as considered as a risk factor for COVID-19. [1]
Rural residence	Using 2016 Census data, this variable was defined as a residence that is not within the commuting zone of a population centre of greater than 10,000 people. We matched information about rural areas to postal codes. We included this variable as a potential marker for access to COVID-19 testing.
Public Health Region	The information in this variable were taken from Public Health Unit (PHU) information from the Registered Persons Database. We defined the regions as follows:<br>Central East PHUs:<br>35 (Haliburton, Kawartha, Pine Ridge District Health Unit),<br>55 (Peterborough County—City Health Unit)<br>60 (Simcoe Muskoka District Health Unit)<br>Central West PHUs:<br>27 (Brant County Health Unit),<br>34 (Haldimand-Norfolk Health Unit),<br>36 (Halton Regional Health Unit),<br>37 (City of Hamilton Health Unit),<br>46 (Niagara Regional Area Health Unit),<br>65 (Waterloo Health Unit),<br>66 (Wellington-Dufferin-Guelph Health Unit)<br>Durham PHUs:<br>30 (Durham Regional Health Unit)<br>Eastern PHUs:<br>38 (Hastings and Prince Edward Counties Health Unit),<br>41 (Kingston, Frontenac and Lennox and Addington Health Unit),<br>43 (Leeds, Grenville and Lanark District Health Unit),<br>57 (Renfrew County and District Health Unit),<br>58 (The Eastern Ontario Health Unit)<br>North PHUs:<br>26 (The District of Algoma Health Unit),<br>47 (North Bay Parry Sound District Health Unit),<br>49 (Northwestern Health Unit),<br>56 (Porcupine Health Unit),<br>61 (Sudbury and District Health Unit),<br>62 (Thunder Bay District Health Unit),<br>63 (Timiskaming Health Unit)<br>Ottawa PHUs:<br>51 (City of Ottawa Health Unit)<br>Peel PHUs:<br>PHU 53 (Peel Regional Health Unit)<br>South West PHUs:<br>31 (Elgin-St. Thomas),<br>33 (Grey Bruce Health Unit),<br>39 (Huron County Health Unit),<br>40 (Chatham-Kent Health Unit),<br>42 (Lambton Health Unit),<br>44 (Middlesex-London Health Unit),<br>52 (Oxford),<br>54 (Perth District Health Unit),<br>68 (Windsor-Essex County Health Unit)<br>Toronto PHU:<br>95 (City of Toronto Health Unit)<br>York PHU:<br>70 (York Regional Health Unit)

© 2022 Hosseini-Moghaddam SM et al. *JAMA Network Open.*

**Variable**	**Definition**
Asthma	We used ICES-specific asthma database to identify patients with asthma based on 2 or more ambulatory care visits and/or 1 or more hospital admissions. This variable was included *a priori* considering its relationship to severe COVID-19 outcomes. [2]<br><u>OHIP</u><br>OHIP diagnostic code: 493<br><u>DAD</u><br>ICD-9 diagnostic code: 493 ICD-10 diagnostic codes: J45, J46
Chronic obstructive pulmonary disease (COPD)	We used ICES-specific COPD database to identify patients with COPD based on 1 or more ambulatory care visits and/or 1 or more hospital admissions. This variable was included *a priori* considering its relationship to severe COVID-19 outcomes.[3]<br><u>OHIP</u><br>OHIP diagnostic codes: 491, 492, 496<br><u>DAD</u><br>ICD-9 diagnostic codes: 491, 492, 496 ICD-10 diagnostic codes: J41, J42, J43, J44
Hypertension	We used ICES-specific hypertension database to identify patients with hypertension, based on 1 or more DAD diagnoses or 2 or more OHIP diagnoses in a two-year period; or 1 OHIP diagnosis followed by an OHIP/DAD diagnosis within two years. This variable was included *a priori* as hypothesized to be directly related to severe COVID-19.[4]<br><u>DAD, SDS:</u><br>ICD-9 diagnostic codes: 401, 402, 403, 404, 405 ICD-10 diagnostic codes: I10, I11, I12, I13, I15<br><u>OHIP:</u><br>OHIP diagnostic codes: 401, 402, 403, 404, or 405
Diabetes mellitus	We used ICES-specific diabetes database to identify patients with diabetes, based on 2 OHIP diagnostic codes or 1 OHIP service code or 1 DAD admission within 2 years. This variable was included *a priori* as hypothesized to be directly related to COVID-19 infection risk. [2]<br><u>OHIP</u><br>OHIP diagnostic code: 250<br>OHIP service codes: Q040, K029, K030, K045, K046<br><u>DAD, SDS</u><br>ICD-9 diagnostic code: 250<br>ICD-10 diagnostic codes: E10, E11, E13, E14
History of congestive heart failure (CHF)	We used ICES-derived CHF database to identify patients with CHF, based on 1 NACRS, DAD, SDS, or OHIP claim and a second claim (from either) in 1 year. The CHF database is limited to those aged 40 years or older. This variable was included *a priori* as hypothesized to be directly related to severe COVID-19.[5]<br><u>OHIP:</u><br>OHIP diagnostic code: 428<br><u>DAD, SDS:</u><br>ICD-9 diagnostic code: 428<br>ICD-10 diagnostic codes: I500, I501, I509
Dementia/Frailty	We used ICES Dementia cohort. In this cohort, patient with dementia are defined as 1 hospitalization for dementia and/or 3 ambulatory visits for dementia, each separated by at least 30 days, within 2 years and/or 1 prescription from ODB. This variable was included *a priori* as hypothesized to be related to be directly related to COVID-19 and severity of illness. [6]<br><u>OHIP definition:</u><br>OHIP diagnostic codes: 290, 331<br><u>DAD, SDS definition:</u><br>ICD-9 diagnostic codes: 0461, 290.0, 290.1, 290.2, 290.3, 290.4, 294, 331.0, 331.1, 331.5<br>ICD-10 diagnostic codes: F00, F01, F02, F03, G30<br><u>ODB definition:</u><br>1 prescription for a cholinesterase inhibitor<br><u>Frailty:</u><br>We used Hospital Frailty Risk Score based on an algorithm already derived using all DAD hospitalizations in the 5 years before index. This risk score also includes frailty-related conditions such as dementia and other chronic conditions that are separately reported.

© 2022 Hosseini-Moghaddam SM et al. *JAMA Network Open.*

Variable	Definition
Cancer	Ontario Cancer Registry was used to identify patients with underlying cancer diagnosed in the 5 years prior to index, except for non-melanoma skin cancer (ICD-O-3 Topography = C44 and Morphology = 87xx3).<br>This variable was included *a priori* as hypothesized to be directly related to COVID-19 infection risk and severity of illness. [7]
Chronic kidney disease (CKD)	This variable was included *a priori* as hypothesized to be directly related to COVID-19 infection risk[8]. We defined this variable as having a CKD diagnosis code in DAD, NACRS, OHIP in the past 5 years, or:<br>o At least 1 dialysis code in each of the 3 months prior to index<br>o Diagnosis and procedure codes found in concept dictionary<br><u>OHIP</u><br>OHIP diagnostic codes: 403, 585<br><u>NACRS, DAD</u><br>ICD-10 diagnostic codes: E102, E112, E132, E142, I12, I13, N08, N18, N19<br>Patients who were on chronic dialysis in the year before index date were identified as those with at least 2 of any of the following codes in OHIP, DAD, or SDS separated by at least 90 days, but less than 150 days.<br><u>OHIP</u><br>OHIP service codes: R849, G323, G325, G326, G860, G862, G865 G863, G866, G330, G331, G332, G333, G861, G082, G083, G085, G090, G091, G092, G093, G094, G095, G096, G294, G295, G864, H540, H740<br><u>DAD, SDS</u><br>CCI procedure codes: 5195, 6698 CCP procedure code: 1PZ21
Immunocompromised (HIV, transplant, immunosuppressive therapy)	We included immunosuppressive conditions *a priori* as hypothesized to be directly related to COVID-19 infection risk[9].<br><br><u>HIV</u><br>We used ICES-specific HIV database to identify patients with HIV, based on 3 physician claims in 3 years with OHIP diagnostic codes: 042, 043 or 044<br><u>CORRLINK</u><br>CORRLINK is an ICES-specific database which links CORR and DAD data. This database only includes patients that have received an organ transplant and does not include dialysis patients.<br>History of allogenic/autologous bone marrow transplant (DAD, OHIP): We identified those who had a history of allogenic bone marrow transplant before index date using the following combination of diagnostic codes:<br><br><u>DAD:</u><br>• Before 2002: CCP code (prcode) = 53.0<br>• 2002 and onwards: CCI code (incode) = 1WY19, 1LZ19HHU7, 1LZ19HHU8<br><br><u>OHIP:</u><br>Feecode = Z426<br>Any hospitalization (any diagnosis field) with the following codes:<br>Sickle-cell disease (ICD10 D57.0 – D57.2; D57.8 OR ICD9 282.6);<br>Hereditary immunodeficiency (ICD10 D80-D84; D89.8, D89.9 and ICD9 279);<br>Neutropenia (ICD10 D70; ICD9 288.0);<br>Functional disorders of polymorphonuclear neutrophils & genetic anomalies of leukocytes (ICD10 D71-D72; ICD9 288.1, 288.2);<br>Hyposplenism, hypersplenism and chronic congestive splenomegaly (ICD10 D73.0, D73.1, D73.2; ICD9 289.4 or 289.5);<br>Asplenia (ICD9 759.0; ICD10 Q89.0);<br>HIV positive (ICD9 042; ICD10 B20) or identified in HIV database

© 2022 Hosseini-Moghaddam SM et al. *JAMA Network Open.*

Variable	Definition
Advanced liver disease (Cirrhosis or Decompensated Cirrhosis)	We included advanced liver disease *a priori* as hypothesized to be directly related to COVID-19 infection risk[10].<br>Defined using the Cirrhosis Algorithm 9:Two or more physician visits (diagnosis code 571), or one or more hospital diagnosis of cirrhosis, using the following diagnostic codes:<br><u>ICD-9</u> : 456.1, 571.2, 571.5<br><u>ICD-10</u>: I85.9, I98.2, K70.3,K71.7, K74.6<br>Defined using the Decompensated Cirrhosis Algorithm 5 (from above reference):<br>One or more physician visits with diagnosis code 571 and (one or more hospital diagnosis or one or more procedure), using the following diagnostic codes:<br><u>ICD-9:</u> 456.0, 456.2, 572.2, 572.3, 572.4, 782.4, 789.5<br><u>ICD-10:</u> I85.0, I86.4, I98.20, I98.3, K721, K729, K76.6, K76.7, R17, R18<br><u>CCI:</u> 1.NA.13.BA-FA, 1.NA.13.BA-X7, 1.NA.13.BA-BD, 1.KQ.76GP-NR, 1.OT.52.HA<br><u>CCP:</u> 1006, 6691<br><u>OHIP:</u> J057, Z591
History of cardiac ischemia	History of cardiac ischemia was included *a priori* as hypothesized to be directly related to COVID-19 infection risk[11].<br><u>Cardiac ischemic disease</u> (DAD, SDS): Any comorbidity in the past 5 years (DAD, any diagnosis field) or history of procedure in past 20 years (DAD, SDS):<br><u>Comorbidity</u> (DAD, any diagnosis in the past 5 years): Angina: ICD-10 diagnostic codes: I20 ICD-9: 413<br><u>Chronic Ischemic Heart Disease</u>: ICD-10 diagnostic codes: I25 ICD-9: 4140, 4148, 4149<br><u>Myocardial infarction</u>: ICD-10 diagnostic codes: I21, I22 ICD-9: 410, 411, 412<br><u>Procedure</u> (DAD & SDS): Coronary Artery Bypass Grafting: CCI procedure codes: 1IJ76 CCP procedure codes: 481<br><u>Percutaneous Coronary Intervention</u>: CCI procedure codes: 1IJ50, 1IJ5, 1IJ57 CCP procedure codes: 4802, 4803
History of transient ischemic attack or acute ischemic stroke	Acute ischemic stroke or transient ischemic attack were included *a priori* as hypothesized to be directly related to COVID-19 infection risk[12].<br><u>Transient Ischemic Attack:</u><br>DAD and NACRS were used to identify patients with a history of a transient ischemic attack, based on at least 1 hospitalization or ED visit with a diagnosis coded with one of the following codes:<br>ICD-9 diagnostic codes: 435, 3623<br>ICD-10 diagnostic codes: G450, G451, G452, G453, G458, G459, H340<br><u>Acute Ischemic Stroke:</u><br>DAD was used to identify patients with a history of acute ischemic stroke, based on at least 1 hospitalization with a main diagnosis coded with one of the following codes:<br>ICD-9 diagnostic codes: 43301, 43311 43321 43331 43381 43391 434, 436<br>ICD-10 diagnostic codes: I63, I64, H34.1
ADG quintiles	ADGs are clusters of similar diagnoses, defined based on their estimated impact on health services resource consumption. Membership in an ADG was calculated by the Johns Hopkins Adjusted Clinical Groups (ACG) software, ACG® System Version 10, based on DAD and OHIP records in the past 2 years. Individuals were ranked by quintile based on the number of ADGs they belonged to.<br>Number of ADGs assigned by Johns Hopkins Adjusted Clinical Groups (ACG) System Version 10 software, using DAD and OHIP records in the 2 years pre-index. We ranked individuals in the province by number of ADGs into 5 categories (quintiles), each containing approximately 20% of the overall Ontario population. There are a total of 32 ADGs that can be defined by the software: "Time Limited: Minor", "Time Limited: Minor- Primary Infections", "Time Limited: Major", "Time Limited: Major-Primary Infections", "Allergies", "Asthma", "Likely to Recur: Discrete", "Likely to Recur: Discrete-Infections", "Chronic Medical: Stable", "Chronic Medical: Unstable", "Chronic Speciality: Stable-Orthopedic", "Chronic Specialty: Stable-Ear, Nose, Throat", "Chronic Specialty: Stable-Eye", "Chronic Specialty: Unstable-Orthopedic", "Chronic Specialty: Unstable-Ear, Nose, Throat", "Chronic Specialty: Unstable-Eye", "Dermatologic", "Injuries/Adverse Effects: Minor", "Injuries/Adverse Effects: Major", "Psychosocial: Time Limited, Minor", "Psychosocial: Recurrent or Persistent, Stable", "Psychosocial: Recurrent or Persistent, Unstable", "Signs/Symptoms: Minor", "Signs/Symptoms: Uncertain", "Signs/Symptoms: Major", "Discretionary", "See and Reassure", "Prevention/Administrative", "Malignancy", "Pregnancy", and "Dental".

© 2022 Hosseini-Moghaddam SM et al. *JAMA Network Open.*

Variable	Definition
Hospital admissions	DAD/OMHRS: Number of acute-care hospital admissions in the 3 years before the index date.
ICU admission	The following databases have been used to detect patients who were admitted to ICU following COVID-19 diagnosis[13]: SCU,CCP,OHIP<br>CCI
Outpatient visits, last year	OHIP: Number of physician office, long-term care, or home visits in the 1 year before the index date[6].
Influenza vaccine received	OHIP billing codes<br>or ODB billing with Drug Identification Numbers (DINs)
Household density quintile	Average number of persons in private households, calculated at the DA level using the 2016 Census data. DAs across the province were ranked by average number of persons per household into 5 categories (quintiles), such that each group contained approximately one-fifth of the DAs.
Apartment building density grouping	Calculated at the DA level using 2016 Census data. This variable was computed by identifying the number of individuals reporting living in an "apartment in a building that has five or more storeys" or "apartment in a building that has fewer than five storeys" and dividing this value by the total number of individuals having answered questions about occupied private dwellings by structural type of dwelling. This yielded a percentage of dwellings in each DA considered to be apartment buildings. DAs across the province were then ranked by these percentages into three groups with cut-offs at the 60th and 80th percentiles, due to a zero-inflated distribution of DAs.
Household income quintile	Calculated at the DA level using 2016 Census data by multiplying the median income (before-tax) by the number of households and dividing by the sum of single-person equivalent to obtain income per single person equivalent. For DAs where median income was unavailable, neighbouring DAs were used to estimate income per single person equivalent. DA- based income quintiles were constructed separately for each census metropolitan area or census agglomeration (one or more adjacent municipalities integrated via commuting flows). DAs within each such area were ranked from the lowest average income per single-person equivalent to the highest, and DAs were assigned to five groups, such that each group contained approximately one-fifth the total in-scope population of each area.
Recent immigration grouping	Calculated at the DA level, using 2016 Census data. This value was obtained by identifying the number of individuals in a DA that reported having immigrated in the past 5 years and dividing this number by the number of individuals in the DA who reported their immigration status over the past five years (i.e., were immigrants or non-immigrants, regardless of the time of immigration).<br>DAs across the province were then ranked by percentage of recent percentages into three groups with cut-offs at the 60th and 80th percentiles, due to a zero-inflated distribution of DAs.

© 2022 Hosseini-Moghaddam SM et al. *JAMA Network Open.*

eReferences

1. Sundaram ME, Calzavara A, Mishra S, et al. Individual and social determinants of SARS-CoV-2 testing and positivity in Ontario, Canada: a population-wide study. CMAJ **2021**; 193:E723–E734. Available at: http://www.ncbi.nlm.nih.gov/pubmed/33906966. Accessed 16 January 2022.
2. Zhang J-J, Dong X, Cao Y-Y, et al. Clinical characteristics of 140 patients infected with SARS-CoV-2 in Wuhan, China. Allergy
3. Chronic obstructive pulmonary disease group of Chinese Thoracic S, Chronic obstructive pulmonary disease committee of Chinese Association of Chest P. Medical management and prevention instruction of chronic obstructive pulmonary disease during the coronavirus disease 2019 epidemic. Zhonghua Jie He He Hu Xi Za Zhi 43:E034–E034. Available at: https://pubmed.ncbi.nlm.nih.gov/32153171 NS -.
4. Wu C, Chen X, Cai Y, et al. Risk Factors Associated With Acute Respiratory Distress Syndrome and Death in Patients With Coronavirus Disease 2019 Pneumonia in Wuhan, China. JAMA Intern Med Available at: http://www.ncbi.nlm.nih.gov/pubmed/32167524 NS -.
5. Chen T, Wu D, Chen H, et al. Clinical characteristics of 113 deceased patients with coronavirus disease 2019: retrospective study. BMJ 368:m1091–m1091. Available at: http://www.bmj.com/lookup/doi/10.1136/bmj.m1295 http://dx.doi.org/doi:10.1136/bmj.m1091%0Ahttp://www.bmj.com/lookup/doi/10.1136/bmj.m1091 NS -.
6. Rosella LC, Kornas K, Bornbaum C, et al. Population-Based Estimates of Health Care Utilization and Expenditures by Adults During the Last 2 Years of Life in Canada's Single-Payer Health System. JAMA Netw open **2020**; 3:e201917. Available at: http://www.ncbi.nlm.nih.gov/pubmed/32236531. Accessed 16 January 2022.
7. Jordan RE, Adab P, Cheng KK. Covid-19: risk factors for severe disease and death. BMJ 368:m1198–m1198. Available at: http://www.bmj.com/lookup/doi/10.1136/bmj.m1198 NS -.
8. Fu D, Yang B, Xu J, Mao Z, Zhou C, Xue C. COVID-19 Infection in a Patient with End-Stage Kidney Disease. Nephron Available at: NS -.
9. Fung, M, Babik JM. COVID-19 in Immunocompromised Hosts: What We Know So Far. Clin Infect Dis **2020**; Available at: https://academic.oup.com/cid/advance-article/doi/10.1093/cid/ciaa863/5864040. Accessed 28 June 2020.
10. Sharma S, Elhence A, Vaishnav M, Kumar R, Shalimar. COVID-19 in patients with cirrhosis: understanding adverse impact. Gut **2021**; 70:1409. Available at: http://www.ncbi.nlm.nih.gov/pubmed/32826307. Accessed 16 January 2022.
11. Peng YD, Meng K, Guan HQ, et al. Clinical characteristics and outcomes of 112 cardiovascular disease patients infected by 2019-nCoV. Zhonghua Xin Xue Guan Bing Za Zhi 48:E004–E004. Available at: https://pubmed.ncbi.nlm.nih.gov/32120458 NS -.
12. Martí-Fàbregas J, Guisado-Alonso D, Delgado-Mederos R, et al. Impact of COVID-19 Infection on the Outcome of Patients With Ischemic Stroke. Stroke **2021**; 52:3908–3917. Available at: https://www.ahajournals.org/doi/10.1161/STROKEAHA.121.034883. Accessed 16 January 2022.
13. Scales DC, Guan J, Martin CM, Redelmeier DA. Administrative data accurately identified intensive care unit admissions in Ontario. J Clin Epidemiol **2006**; 59:802–807. Available at: http://www.ncbi.nlm.nih.gov/pubmed/16828673. Accessed 11 May 2020.

© 2022 Hosseini-Moghaddam SM et al. *JAMA Network Open.*
